# Editorial: Inequities and disparities in reproductive health: reproductive epidemiology

**DOI:** 10.3389/frph.2024.1419178

**Published:** 2024-05-13

**Authors:** Julia D. López, Intira Sriprasert, Melissa Lee Wilson

**Affiliations:** ^1^ Washington University School of Medicine, St. Louis, MO, United States; ^2^ Keck School of Medicine, University of Southern California, Los Angeles, CA, United States

**Keywords:** reproductive health disparities, social determinants of health, maternal mortality and morbidity, pregnancy and postpartum care, epidemiology

**Editorial on the Research Topic** Inequities and disparities in reproductive health: reproductive epidemiology

## Introduction

According to the World Health Organization (WHO), reproductive health encompasses holistic well-being, which includes physical, mental, and social health, and is not merely based on the absence of disease (1). Further, health disparities refer to differences in health outcomes and access to care between various groups. For example, such differences can be influenced by gender, age, sexual identity, race/ethnicity, and socioeconomic status (SES), which impact access to healthcare services and quality of care among various groups (2). Health inequity goes beyond disparities and refers to unjust differences in health outcomes between different groups, rooted in economic, social, and environmental injustices that lead to unequal access to resources, opportunities, and power (3).

The WHO initiative on sexual and reproductive health aims to assess social determinants of health (SDOH) related to maternal mortality and morbidity by supporting high-quality research to strengthen research capacity in low- and -middle-income (LMIC) settings and inform the WHO of norms and standards about SDOH (4). Despite the effort to improve access and utilization of reproductive health services across the globe, reflecting WHO policy, there remain inequities and disparities in reproductive health across social determinants (2, 5), according to race/ethnicity (2, 6), SES (7), culture (8), and politically-influenced policies (9, 10). In addition, the American College of Obstetricians and Gynecologists (ACOG) stated in their committee opinion that recognizing the importance of SDOH can help healthcare providers better understand patients, effectively communicate about health-related conditions and behavior, and improve health outcomes (11).

The social determinants of adverse reproductive outcomes vary across people, place, and time; thus, what and how to measure social determinants varies across communities, societies, countries, and cultures. Therefore, interventions must be curated to the intended population. For example, in LMIC, factors that increase the risk of maternal morbidity and mortality include adolescent pregnancy, primigravidity, nutritional deficiencies, limited education levels, and refugee status (12). Sheikh et al. have suggested some potential interventions in these communities to improve outcomes in pregnant people, such as supplementation with iron and calcium, community-based educational programs, financial incentives to obtain adequate prenatal care, and interventions directed toward the promotion of contraceptive use (12). Additionally, cultural competency among healthcare providers is an essential component of addressing disparities in reproductive health (13). Educational activities should include didactics addressing health literacy, access to health care, and unconscious bias. From the pregnant patient perspective, Brito et al. found that patients feel increased medical education on SDOH would help address factors that lead to disparities in antenatal health care.

Global programs and activities, such as the UN Millennium Development Goals (MDGs), Global Strategy for Women's, Children's, Adolescents’ Health, and the WHO Global Action Plan aim to improve women's health worldwide. While some progress has been made, life expectancy for women remains lower in LMIC, and maternal mortality remains high (14). In contrast, factors impacting women's health in more economically developed countries (MEDC) include younger age, lower socioeconomic attainment, lack of connection with the social environment, and adverse life events (15).

This Special Issue covers several aspects of reproductive health, with a focus on inequities and disparities regarding (1) general reproductive health support and accessibility, (2) factors associated with pregnancy and childbirth, and (3) postpartum effects on the health of women and their children.

## General reproductive health support and accessibility

In many countries, there remains an unsolved issue of general reproductive health support, which is a fundamental human right (16). Factors such as race/ethnicity, access to infrastructure to ensure hygiene for menstruation among sex workers (Phillips-Howard et al.), access to reproductive health services among migrants (Panchenko et al.), the tendency for marginalized individuals not to receive sufficient medical education, and public health policy that often discriminates against marginalized individuals play a part in maintaining inaccessibility (2). A recent study by Panchenko et al. highlights the challenges migrants face, such as poor gynecological care, an absence of dedicated services for pregnant people, and the general lack of reporting systems as it relates to sexual violence and exploitation experienced during the migration journey. When considering the context that pregnant people are faced with, these factors can exacerbate feelings of helplessness and amplify vulnerability in accessing care, which can lead to unequal reproductive health services.

## Factors associated with pregnancy and childbirth

Inadequate sex education and access to contraception persist, which contributes to the ongoing prevalence of unintended pregnancies; therefore, significantly younger age at first birth has been noted, especially in LMIC and in certain ethnicities within MEDC (17). Another study in this Special Issue by Kitaw and Haile found that in Ethiopia the median time to first childbirth was 18 years and that timing of childbirth is associated with educational level, knowledge of contraceptive methods, and exposure to media. The study also noted that increased education and awareness are helpful in reducing disparities in these populations (Kitaw and Haile). Disparities in accessing prenatal care can also contribute to worse outcomes. Stegman et al. have suggested that directed approaches are needed to increase participation in prenatal care, which would likely result in improved outcomes (Stegman et al.). Using the Demographic Health Survey (DHA) from 61 LMICs, Aragaw et al. showed that unintended pregnancies occur at a rate of 26.46%. Factors that were elucidated in this study included media exposure, working status, access to healthcare facilities, and paternal education (Aragaw et al.). Thus, it is critical to use multi-level interventions to support preventative measures to reduce the burden of unintended pregnancies and improve overall reproductive health outcomes.

## Postpartum effects on the health of women and their children

Postpartum education and support regarding nutrition for mothers and children are still limited in some areas, as demonstrated by low rates of breastfeeding and underweight children (18). Interventions are needed to improve nutritional knowledge, attitude, and self-efficacy and reduce the prevalence of underweight children (Chen et al.). Gizaw et al. noted that a positive deviant approach (PDA), whereby the community's strengths are utilized along with problem-solving methodology to empower the community, increased breastfeeding knowledge, attitudes, and self-efficacy.

The articles in this Special Issue demonstrate the pervasive inequities and disparities in reproductive health, underscoring their prevalence across societies worldwide. Addressing these urgent issues requires collaboration from multidisciplinary teams and consideration of location-specific factors to limit unnecessary morbidity and mortality of pregnant people, women, and children.
